# Overexpression of regulator of G protein signaling 11 promotes cell migration and associates with advanced stages and aggressiveness of lung adenocarcinoma

**DOI:** 10.18632/oncotarget.8860

**Published:** 2016-04-20

**Authors:** Sheng-Huei Yang, Chien-Feng Li, Pei-Yi Chu, Hsiu-Hsing Ko, Li-Tzong Chen, Wan-Wen Chen, Chia-Hung Han, Jr-Hau Lung, Neng-Yao Shih

**Affiliations:** ^1^ Joint Biobank, Office of Human Research, Taipei Medical University, Taipei, Taiwan; ^2^ National Institute of Cancer Research, National Health Research Institutes, Tainan, Taiwan; ^3^ Department of Pathology, Chi-Mei Medical Center, Tainan, Taiwan; ^4^ Department of Pathology, Show Chwan Memorial Hospital, Changhua City, Taiwan; ^5^ Division of Infectious Diseases and Tropical Medicine, Department of Internal Medicine, Tri-Service General Hospital, Taipei, Taiwan; ^6^ Division of Pulmonary and Critical care Medicine, Department of Internal Medicine, Chang Gung Memorial Hospital, Chiayi, Taiwan; ^7^ Graduate Institute of Medicine, College of Medicine, Kaoshiung Medical University, Kaoshiung, Taiwan

**Keywords:** RGS11, cell migration, lung adenocarcinoma, MAPK-FAK signalings

## Abstract

Regulator of G protein signaling 11 (RGS11), a member of the R7 subfamily of RGS proteins, is a well-characterized GTPase-accelerating protein that is involved in the heterotrimeric G protein regulation of the amplitude and kinetics of receptor-promoted signaling in retinal bipolar and nerve cells. However, the role of RGS11 in cancer is completely unclear. Using subtractive hybridization analysis, we found that RGS11 was highly expressed in the lymph-node metastatic tissues and bone-metastatic tumors obtained from patients with lung adenocarcinoma. Characterization of the clinicopathological features of 91 patients showed that around 57.1% of the tumor samples displayed RGS11 overexpression that was associated with primary tumor status, nodal metastasis and increased disease stages. Its high expression was an independent predictive factor for poor prognosis of these patients. Cotransfection of guanine nucleotide-binding protein beta-5 (GNB5) markedly increased RGS11 expression. Enhancement or attenuation of RGS11 expression pinpointed its specific role in cell migration, but not in cell invasion and proliferation. Signaling events initiated by the RGS11–GNB5 coexpression activated the c-Raf/ERK/FAK-mediated pathway through upregulation of the Rac1 activity. Consistently, increasing the cell invasiveness of the transfectants by additional cotransfection of the exogenous urokinase–plasminogen activator gene caused a significant promotion in cell invasion *in vitro* and *in vivo*, confirming that RGS11 functions in cell migration, but requires additional proteolytic activity for cell and tissue invasion. Collectively, overexpression of RGS11 promotes cell migration, participates in tumor metastasis, and correlates the clinicopathological conditions of patients with lung adenocarcinoma.

## INTRODUCTION

In the past few decades, studies have shown that G protein-coupled receptors (GPCRs) are highly expressed in cancerous tissues and that their mitogenic ligands are enriched in metastatic sites [1, 2]. The biological consequences of their overexpression are frequently associated with uncontrolled cell growth, survival from apoptotic signals, and development of tumor metastasis. The downstream signaling pathways include activation of phospholipase C, Src, and/or phosphatidylinositol 3 kinase [1, 3]. Hence, regulation of the oncogenic GPCR activities and their downstream signaling is a critical issue for a better understanding of the molecular basis of cancer and for further developments in the diagnosis and treatment of cancer.

The regulator of G protein signaling (RGS) family proteins function as guanosine triphosphatase (GTPase)-accelerating proteins (GAP) that terminate Gα-coupled GPCR signaling rapidly through stabilization of the transition state during the hydrolysis of GTP by Gα [4]. RGS comprises diverse protein families whose members each have a unique tissue distribution, which is strongly related to signal transduction events. About 30 members have been characterized, and all contain at minimum a conserved 120-amino acid domain, known as an “RGS box” [2]. Many RGS proteins also contain multiple structural and functional motifs through which they mediate crosstalk between GPCR-dependent and -independent signaling pathways. Although aberrant G protein activation has been linked to the initiation and progression of various cancers [2, 5], the role of RGS proteins in tumor metastasis remains obscure.

One RGS protein, RGS4, plays a role in controlling cancer cell invasiveness. Weiler *et al.* identified RGS4 as a novel target of CCI-779, a mammalian target of rapamycin (mTOR) inhibitior. Blockade of RGS4 by CCI-779 markedly suppresses glioma cell invasion, suggesting that RGS4 is a key driver of glioblastoma invasiveness [6]. Increased RGS17 expression has been detected in prostate cancer, and knockdown of its expression also results in decreased proliferation of other cancer cells [7]. Additionally, RGS2 is downregulated in prostate cancer [8] and acute myeloid leukemia [9], but RGS5 is upregulated in hepatocellular [10], breast, and ovarian carcinomas [11]. However, few studies have focused on the role of the R7 subfamily of RGS (R7 RGS) proteins in cancer.

The physiological roles of the R7 RGS family in regulating fundamental neural functions by increasing GTP hydrolysis of a selective subset of Gα and modulating GPCR-mediated cellular responses are well documented. This subfamily comprises four homologous proteins, RGS6, RGS7, RGS9, and RGS11, which are highly expressed in the nervous system and share some common multidomains. Heterodimerization of R7 RGS proteins with guanine nucleotide-binding protein beta-5 (GNB5) is indispensable for their protein stability and biological functions in the regulation of synaptic transmission, vision, and postnatal development [12–14]. By contrast, only a few reports have disclosed the pathogenic roles of R7 RGS proteins in cancers. Hurst's group [2] demonstrated an inhibitory role of RGS6 in lysophosphatidic acid-stimulated growth in ovarian cancer cells. A study of single-nucleotide polymorphism of RGS7 showed a significant association with the overall survival of lung cancer patients treated with chemoradiotherapy [15]. Increased expression of RGS11 is shown to be associated with oxaliplatin resistance in colorectal cancer [16]. However, the mechanisms underlying the regulation of cancer by R7 RGS proteins remain unexplored.

Using subtractive hybridization analysis of two pairs of primary lung adenocarcinoma and their metastatic tumor counterparts in lymph nodes (LNs), we found that RGS11 was highly overexpressed in lung metastatic adenocarcinoma, and its overexpression was associated with poorer prognosis, as reflected in shorter disease-free and metastasis-free survivals. In present study, we demonstrate that increased expression of RGS11 can lead to promotion of Rac1-dependent cell migration through activation of the c-Raf–extracellular signal-regulated kinase (ERK)–focal adhesion kinase (FAK) signaling linkage.

## RESULTS

### Overexpression of RGS11 in lung metastatic adenocarcinoma

**Figure 1:**
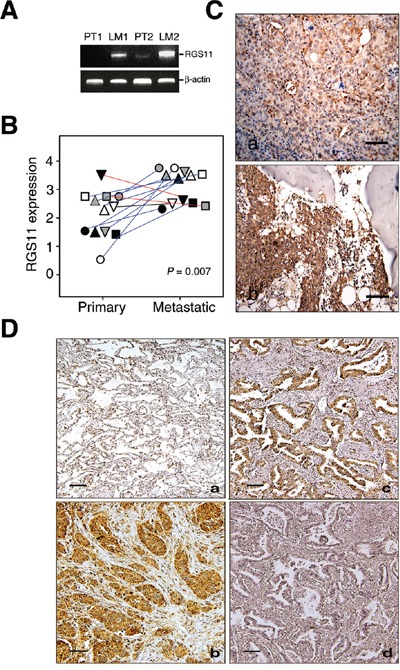
Upregulation of RGS11 was strongly associated with development of metastatic potency in lung adenocarcinoma Two pairs of fresh primary tumor tissues and their LN metastatic counterparts were obtained from two patients with lung adenocarcinoma. **A.** RT-PCR analysis of RGS11 expression. Total RNA was extracted from the tissues and reverse transcribed into cDNA. Putative metastatic genes were enriched by a subtraction hybridization procedure, which included RGS11 in the present study. Its gene expression was analyzed by PCR using primers specific to RGS11 or β-actin control, as indicated [refer to Supplementary Materials and Methods]. The resultant products were resolved in 1% ethidium bromide-containing agarose gel. “PT” and “LM” indicate primary and LN metastatic tumors, respectively. **B.** Quantification and statistical analysis of RGS11 expression in tumor tissues. Twelve pairs of lung primary and bone metastatic counterparts were stained immunohistochemically with RGS11 antibody, and the expression level was scored using a standard pathology determination by two experienced pathologists. Each symbol represents an individual paired sample, and the data show a significant increase in RGS11 expression in the metastatic lesions from 9 of 12 patients (blue line). Decreased or no differential expression of RGS11 in other samples is marked with red or black line, respectively. The differential expression of RGS11 between primary tumors and their metastatic counterparts was analyzed using chi-square analysis (*P* = 0.007). “*P*” represents the statistical significance. **C.** IHC study of RGS11 expression in primary and metastatic tumors. Twelve pairs of lung primary and bone-metastatic tumors were determined by IHC analysis using antibody specific to RGS11. One representative paired primary (C, a) and bone metastatic counterpart (C, b) sample are displayed. **D.** IHC study of lung adenocarcinoma samples. Tissue sections from 91 patients with lung adenocarcinoma and their tumor-adjacent normal counterparts were stained immunohistochemically with RGS11 antibody. (D, a) Normal-appearing alveolar tissues proximal to the tumor showed a low or undetectable immunoreactivity to RGS11. By contrast, increased RGS11 expression in the cytoplasm was found in the tumor cells. Two representative samples are shown here (D, b and c). The specificity of RGS11 antibody was demonstrated by pre-absorbing the antibody with its antigen (D, d). Magnification, 200×. Scale bar, 100 μm.

**Figure 2:**
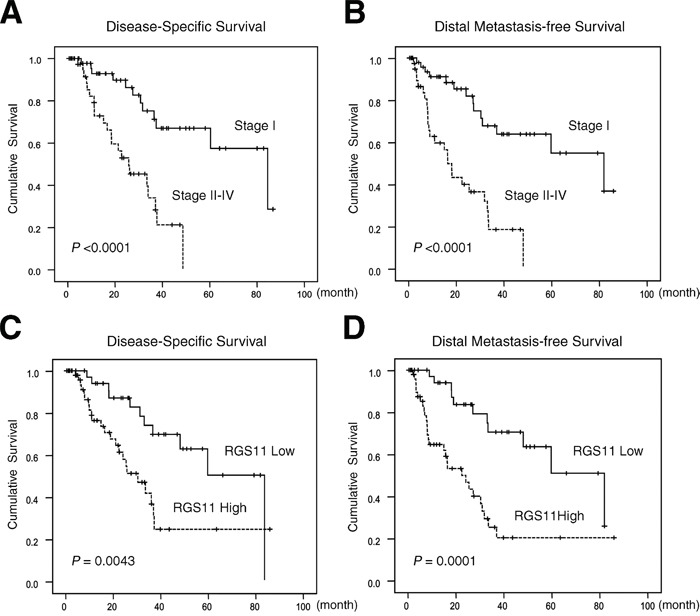
Kaplan–Meier analyses of DSS and DMFS Tissue sections from 91 patients with lung adenocarcinoma were immunostained with the RGS11 antibody. Histological types and tumor grading were determined according to the 2000 WHO classification. Pathology staging of the disease was assigned based on the 7^th^ edition of the AJCC system. DSS and DMFS are expressed according to the disease staging (**A** and **B.** respectively) and according to the pathology staining score for RGS11 expression as ≤2 (Low) or >2 (High) (**C** and **D**). Statistical significances (*P*-values) are indicated on the bottom left.

Tumor metastasis is the major cause of the disease-specific death of patients with lung adenocarcinoma. To identify the genes that might play a pivotal role in metastatic events, two pairs of fresh primary tumors and their LN metastatic counterparts were analyzed. The mRNA was extracted and reversely transcribed into cDNA pools. After subtractive hybridization, the *RGS11* gene was shown by RT-PCR analysis to be highly upregulated in the metastatic tumors as compared with the corresponding primary tumors (Figure 1A). Because of the limited LN tumor samples available, 12 pairs of lung primary and bone metastatic samples were used in the comparison of RGS11 expression in these two types of tumors. The results of the histological examination and scoring by two experienced pathologists (Figure 1B and 1C) showed a significantly increased expression in the metastatic lesions from 9 of 12 patients (*P* = 0.007). In addition, to determine whether the expression status of RGS11 correlates with disease progression, 91 lung adenocarcinoma samples were analyzed. Histological examination in Figure 1D demonstrates that RGS11 was detected primarily in the cytoplasm of the tumor cells, but its expression was low or absent in the tumor-adjacent normal lung tissues. Around 57.1% (52/91) of the samples displayed moderate or strong RGS11-positive immunoreactivity in no less than 50% of the tumor cells. Characterization of the clinicopathological features of the patients indicated that RGS11 overexpression was significantly associated with increased primary tumor status, nodal metastasis, and disease stages, but not related to gender or age (Table 1). Univariate log-rank analysis was used to quantify the disease-specific survival (DSS) and distal metastasis-free survival (DMFS). A more aggressive clinical course and significantly shorter DSS and DMFS were observed in patients with nodal metastasis (*P* < 0.0001 for both end points), higher disease stage (*P* < 0.001 for both end points), and RGS11 overexpression (*P* = 0.0043 and *P* = 0.0001 for DSS and DMFS, respectively) (Table 2). Notably, as shown in Table 3 and Figure 2, RGS11 overexpression was both a univariate predictor for worse outcomes and an independent indicator of poor DSS (*P* = 0.0274, risk ratio [RR] = 2.318) and DMFS (*P* = 0.0010, RR = 3.313). Taken together, data support the idea that RGS11 overexpression is strongly associated with the aggressiveness of lung adenocarcinoma.

**Table 1:** Association of RGS11 expression with clinicopthologic variables

RGS11 expression				
Parameter	Total (%)	Low expression Score ≦ 2 (%)	High expression Score > 2 (%)	*P*-value
Age (year old)				
<60	22 (24.2)	9 (40.9)	13 (59.1)	0.832
≦60	69 (75.8)	30 (43.5)	39 (56.5)	
Gender				
Male	44 (48.4)	20 (45.5)	24 (54.5)	0.628
Female	47 (51.6)	19 (40.5)	28 (59.5)	
Primary tumor (T)				
T1	35 (38.5)	20 (57.1)	15 (42.9)	0.029*
T2-T4	56 (61.5)	19 (33.9)	37 (66.1)	
Nodal status (N)				
N0	51 (56.0)	27 (52.9)	24 (47.1)	0.028*
N1-N2	40 (44.0)	12 (30.0)	28 (70.0)	
Disease stage				
I	49 (53.8)	26 (53.1)	23 (46.9)	0.034*
II-IV	42 (46.2)	13 (31.0)	29 (69.0)	

Note: The associations between various clinicopathological parameters and RGS11 expression status were evaluated using the chi-square test.

* represents statistical significance, *P* < 0.05.

**Table 2:** Univariate log-rank analyses for disease-specific survival (DSS) and distal-metastasis free survivals (DMFS)

Parameters	Case No.	DSS		DMFS	
		Event No.^#^	*P* -value	Event No.^#^	*P* -value
Gender					
Male	44	14	0.8312	17	0.9887
Female	47	20		23	
Age (year old)					
< 60	22	7	0.3138	10	0.8714
≦60	69	67		30	
Primary tumor (T)					
T1	35	11	0.0705	12	0.0295*
T2-T4	56	23		28	
Nodal status (N)					
N0	51	13	<0.0001***	15	<0.0001***
N1-N2	40	21		25	
Disease stage					
I	49	12	<0.0001***	14	<0.0001***
II-IV	42	22		26	
RGS11 expression					
Low ≦ 2	39	11	0.0043* *	11	0.0001***
High > 2	52	23		29	

Notes:

# indicates number of patients who developed disease-specific or distal-metastatic death for DSS or DMFS, respectively. Univariate log-rank analysis was used to quantify DSS and DMFS. “*”, “**”, and “***” represent statistical significance, *P* < 0.05, *P <* 0.01, and *P* < 0.001, respectively.

**Table 3:** Multivariate survival analyses

Parameter	DSS			DMFS		
	H.R	95% CI	*P*-value	H.R	95% CI	*P*-value
Stage						
I	1.0	1.76-8.31	0.0007***	1.0	2.03-8.28	<0.0001***
II-IV	3.83	-		4.10	-	
RGS11 Expression						
Low ≦ 2	1.0	1.10-4.89	0.0274*	1.0	1.62-6.77	0.0010**
High > 2	2.32	-		3.31	-	

Notes: A multivariate model using Cox proportional-hazards regression included the parameters with a univariate *P*-value < 0.05; “*”, “**”, and “***” represent statistical significance, *P* < 0.05, *P* < 0.01, and *P* < 0.001, respectively.

### Promotion of cell migration by RGS11

**Figure 3:**
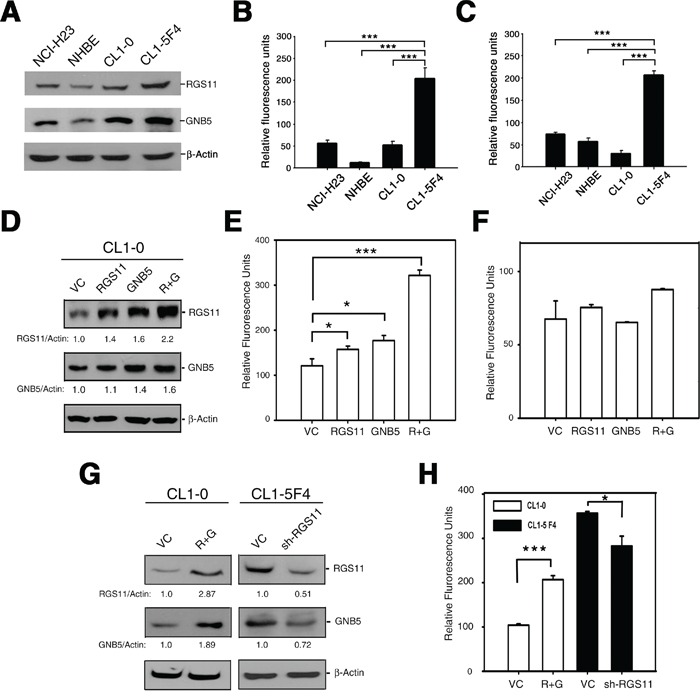
Association of RGS11 expression status with cell migration potency **A.** Endogenous level of RGS11 and GNB5. The cell lysates (50 μg/cell line) of NCI-H23, NHBE, CL1-0, and CL1-5F4 cells were resolved by SDS-PAGE, and the bands were immunoblotted with antibody specific to RGS11 or GNB5, as indicated on the right. NHBE cells served as a normal lung cell control, and β-Actin as a protein loading control. **B-C.** Transwell cell migration and invasion assays. Cells (5 × 10^5^ / reaction) were starved in serum-free medium for 2 h at 37°C, seeded into the upper chamber of the Transwell inserts with 8 μm-pore size polycarbonate membranes uncoated or coated with tumor-associated extracellular matrix and subjected to the migration and invasion assays, respectively. The cells were chemoattracted by medium containing 10% FBS in the bottom chamber at 37°C for 6 h (cell migration) or 24 h (cell invasion). The cells that moved through the membranes were quantified using a fluorometric detection system according to the manufacturer's instruction. The experiments were performed in triplicate, and the data are expressed as the mean ± SD. *** indicates *P* < 0.001. **D.** Exogenous expression of RGS11 and GNB5. CL1-0 cells were transiently transfected with a plasmid encoding for RGS11, GNB5, or the combination of RGS11 and GNB5, as indicated. The expression levels of the corresponding proteins of the transfectants were determined by Western blotting, as described above. The expression levels were normalized to that of β-Actin and are represented as fold increase compared with the control cells (VC), as indicated at the bottom. **E-F.** Effect of gene manipulation on cell migration and invasion. Cell migration and invasion assays were performed in Transwell chambers, as described above. Data are expressed as the mean ± SD of triplicates. **G.** Western blot analysis of RGS11 and GNB5 in stable transfectants. CL1-0 cells were cotransfected with plasmids encoding for RGS11 and GNB5 (R+G), and then treated with G418 and hygromycin. CL1-5F4 stable transfectants with knocked-down endogenous RGS11 expression (sh-RGS11) were selected in medium containing puromycin. The levels of RGS11 and GNB5 expression were determined by Western blotting. **H.** Cell migration of the stable cells. The cell migration capability of the transfectants with overexpressed of RGS11 and GNB5 (R+G) or attenuated expression of RGS11 (sh-RGS11) were determined by the Transwell assay performed as described above. The data were analyzed using Student's *t* test. * and *** indicate *P* < 0.05 and *P* < 0.001, respectively.

To examine the relationship between RGS11 expression status and metastatic potency, the cell migration and invasion capabilities of lung adenocarcinoma cell lines were examined closely using Western blotting and Boyden-chamber transwell assays. After 2 h starvation, the cells were chemoattracted by serum stimulation for another 6 h (cell migration) or 24 h (cell invasion). The results in Figure 3A show that the expression level of RGS11 was related to cell mobility. Highly invasive CL1-5F4 cells, which express a significant amount of RGS11 and its associated partner GNB5, displayed greater cell migration (Figure 3B) and invasion (Figure 3C) activities, as compared with SV40-immortalized human normal bronchial epithelial (NHBE) cells and with low-invasive NCI-H23 and CL1-0 cells. In addition, a similar phenomenon was also observed in aggressive A549 cells, showing that higher expression of RGS11 is positively associated with their migratory and invasive behaviors when compared to those in NHBE cells (Supplementary Figure S1).

To examine the expression profiles of RGS11 and other R7 RGS family members, CL1-5F4 and CL1-0 cells were examined by RT-PCR and Western blot analyses. The expression levels of RGS11 and GNB5 were higher in CL1-5F4 than in CL1-0 cells. The expression of RGS6, RGS7 and RGS9 were much lower or even absent in CL1-5F4 cells when compared with CL1-0 cells (Supplementary Figure S2). These data further support the idea that high expression of RGS11 is involved in cell movement.

Since R7 RGS proteins, including RGS11, could be stabilized by heterodimerization of GNB5 in previous reports [12–14], we further investigated whether decrease in GNB5 association might destabilize the R7 RGS members in CL1-5F4 and A549 cells. Endogenous GNB5 of the cells was attenuated by transfecting small hairpin interfering RNA specific to GNB5 (sh-GNB5). This action obviously decreased the expression of RGS6 and RGS11, but barely affected on RGS7 and RGS9 expression in both cell lines (Supplementary Figure S3). To avoid the complexity of GNB5 with other R7 RGS family members in cell mobility, RGS11 was particularly focused in present study.

To study the specific role of RGS11 in cell movement in detail, its gene expression was manipulated, and cell chemoattractive studies were performed using transwell assays. Consistent with the findings of previous reports [12], RGS11 was stabilized by increasing GNB5 expression in CL1-0 cells (Figure 3D). Transient transfection with RGS11 and/or GNB5 significantly promoted the cell migration (Figure 3E) but not cell invasion (Figure 3F); in particular, cotransfection of both genes. Consistent results were obtained in CL1-0 cells that stably coexpressed RGS11 and GNB5 (R+G) and in CL1-5F4 stable transfectants with attenuated expression of RGS11 induced by small hairpin interfering RGS11 (sh-RGS11) (Figure 3G and 3H). Intriguingly, knockdown of endogenous RGS11 also slightly decreased the GNB5 expression, suggesting that increase in RGS11 expression by association of GNB5 is a key factor in the promotion of cell migration. This finding was also strongly supported by other cell models. Experiment using coexpression of RGS11-GNB5 (R+G) in NCI-H23 cells and attenuated expression of RGS11 (sh-RGS11) in A549 cells consistently demonstrated its major function on cell migration (Supplementary Figure S4B and S4E). In agreement with the CL1-0 study (Figure 3F), increasing expression of RGS11, GNB5, or combination of RGS11-GNB5 (R+G) did not significantly elevate the invasive potency of H23 cells (Supplementary Figure S4C). Decreased expression of RGS11 in A549 causing a severe impairment on cell invasion could be attributed to loss of migratory capability of the cells (Supplementary Figure S4E-S4F). On the other hand, the gene manipulation did not significantly affect cell growth (Supplementary Figure S5). Collectively, data pinpoint a specific role of the RGS11–GNB5 complex in cell migration, but not in cell invasion and cell proliferation.

### Promotion of cell migration by RGS11–GNB5 through activation of mitogen-activated protein kinase (MAPK) and FAK signalings

**Figure 4:**
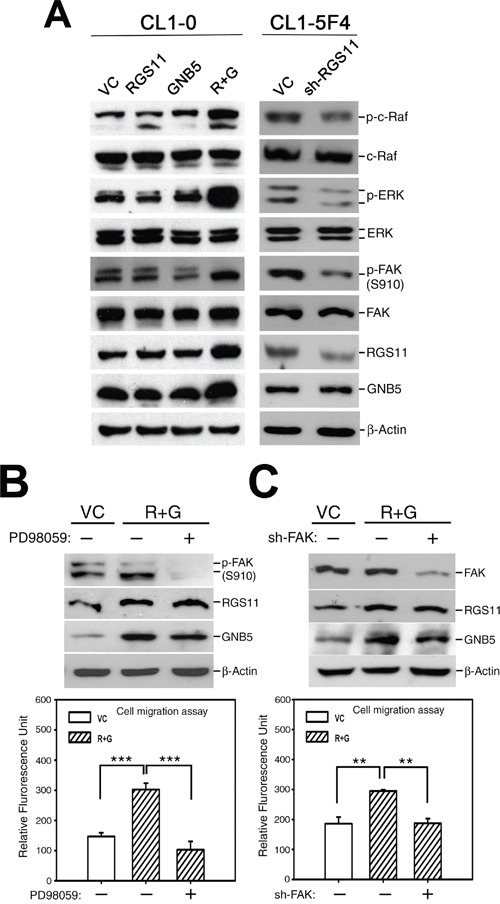
Promotion of cell migration by coupling of RGS11–GNB5 expression with activation of Raf–ERK–FAK signaling **A.** Activation of Raf–ERK–FAK signaling. The cell lysates of CL1-0 transfectants that stably expressed RGS11, GNB5, or combination of RGS11 and GNB5 (R+G) as well as the lysates of CL1-5F4 transfectants with the RGS11-specific small hairpin RNA (shRNA) (sh-RGS11) were resolved by 10% SDS-PAGE, blotted onto a nitrocellulose membrane, and probed with antibody specific to p-c-Raf (Ser338), total c-Raf, p-ERK (Thr202/Tyr204), ERK, p-FAK (Ser910), FAK, RGS11 and GNB5. Control cells were transfected with their corresponding vectors (VC). Beta-Actin (β-Actin) serves as a control. **B.** Inhibition of MAPK/ERK activity. The RGS11–GNB5 transfectants (R+G) were pretreated with 20 μM PD98059 (MAPK/ERK inhibitor) for 2 h or not, as indicated. **C.** Attenuation of FAK expression. The RGS11–GNB5 and control (VC) transfectants were seeded 24 h before treatment. Subsequently, the R+G transfectants were transiently transfected with the FAK-specific shRNA (sh-FAK) for 48 h or not, as indicated. The cell migration capability was measured in *in vitro* Transwell assays for 6 h at 37°C. The experiments were performed in triplicate, and the data are expressed as the mean ± SD. ** and *** indicate *P* < 0.01 and *P* < 0.001, respectively.

To dissect the signaling events mediated by RGS11–GNB5, pharmacological inhibitors were used to block the promotion effect of various signaling pathways on cell migration. Treatment of the CL1-0 transfectants with AG1478, PP2, or PD98059 (EGFR, Src, and MAPK inhibitors, respectively) abolished the biological activity (data not shown). MAPK–FAK signaling linkage has been reported to be their common downstream pathway [3]. Overexpression of RGS11–GNB5 (R+G), indeed, increased c-Raf, ERK, and FAK activities in CL1-0 cells when compared with controls, and vice versa in CL1-5F4 cells (Figure 4A). Blocking MAPK activation by PD98059 1 h before the transwell assay caused marked dephosphorylation of FAK and abolished CL1-0 cell migration (Figure 4B), indicating that MAPK activity is a prerequisite for FAK activation in the RGS11–GNB5 promotion of cell migration. An experiment using exogenous transfection of FAK-specific small hairpin interfering RNA (sh-FAK) to attenuate FAK expression showed clearly that FAK was the major kinase responsible for the cell migration (Figure 4C). These findings suggest that the RGS11–GNB5-mediated signaling cascade shares a similar mechanism with Ras-induced sequential modification of FAK activity through phosphorylation of the Ser910 residue by ERK [17].

### RGS11–GNB5-induced upregulation of Rac activity

**Figure 5:**
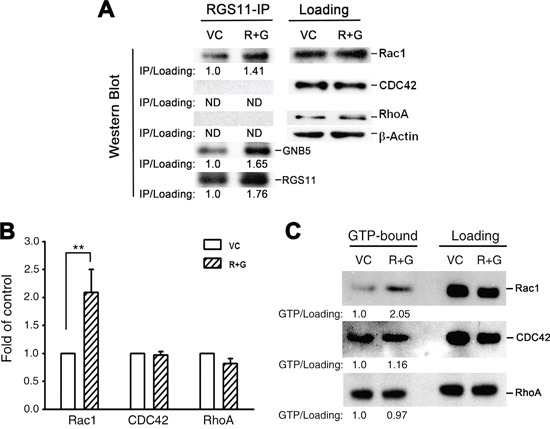
RGS11/GNB5-mediated cell migration via activation of Rac1 CL1-0 stable transfectants (R+G) that overexpressed RGS11 and GNB5 and their vector control (VC) cells were lysed. **A.** Immunoprecipitation and immunoblot. The lysates were pre-cleaned with Protein A/G Sepharose beads, and the protein concentration of supernatants was determined by BCA assay. The pre-cleaned lysates (500 μg/reaction) were incubated with antibody against RGS11 (20 μg/reaction) for 2 h, and followed by addition of 50 μL of the Sepharose beads with gently rocking at 4°C overnight. After wash, the RGS11 precipitants (RGS11-IP) were denatured in boiling Laemmli sample buffer, resolved by SDS-PAGE, and Western blotted with antibody against RGS11, GNB5 or RhoA/Rac1/CDC42 in the G-LISA systems (*left*). Western blotting analysis of the pre-cleaned lysates shows the total levels of Rac1, CDC42, and RhoA (*right*). Beta-Actin (β-Actin) acts as a loading control. The levels of Rac1, CDC42, and RhoA precipitants were first normalized with their corresponding total forms. Precipitated RGS11 and GNB5 were normalized with β-Actin. The data are expressed as the ratio of each precipitant in the R+G transfectants compared with that in the control cells (*left*). “ND” stands for no signal detected. **B.** G-LISA assay. The GTP-bound RhoA, Rac1, and CDC42 activity in the lysates (100 μg/sample) were analyzed by binding to the corresponding GTP-binding protein. The complex was detected with antibodies specific to the active forms using the absorbance-based G-LISA system (Cytoskeleton) at 490 nm. The experiments were performed in triplicate, and the data are expressed as the mean ± SD fold increase compared with the control cells (VC). ** indicates *P* < 0.01. **C.** Pull-down assay. The cell lysates (500 μg/reaction) were prepared. The active forms of RhoA and Rac1–CDC42 were pulled down by glutathione–agarose beads conjugated with GST–Rhotekin–RBD and GST–PAK–PBD, respectively, resolved by 15% SDS-PAGE, and detected with antibodies specific to Rac1, CDC42, and RhoA according to the manufacturer's instructions (Cytoskeleton). The left (GTP-bound) and right (total loading) panels showed the active forms and total amount of each Rho family member, as indicated. The levels of the active forms were first normalized with their corresponding total forms. The data are expressed as the ratio of each active form in the R+G transfectants compared with that in the control cells (below).

To identify the mediator(s) downstream to RGS11–GNB5 required for the progression from signal transmission to MAPK/ERK activation, three small G proteins known to be involved in cell skeleton reorganization and cell migration, Rac1, CDC42, and RhoA, were studied. Since GNB5 is considered as a pivotal mediator integrating signalings between receptors, such as GPCRs, and effectors, such as RhoA and Rac1, in a public protein-protein interaction dataset [32], RGS11 was plausibly speculated to interact with the small G proteins through association of GNB5. To verify this hypothesis, experiment using immunoprecipitation of RGS11 and immunoblotting for GNB5 and individual small G proteins revealed a physical association of RGS11 with GNB5 and Rac1 in CL1-0 stable transfectants, whereas CDC42 and RhoA did not appear in the RGS11 precipitants (Figure 5A). To further investigate whether this protein complex could modulate the activity of Rac1 and other small G proteins in the cells, their corresponding interacting proteins, Rhotekin–RBD and PAK–PBD as well as antibodies specific to individual small G-proteins were used in the G-LISA^®^ system and pull-down assay (Figure 5B and 5C, respectively). Upregulation of RGS11–GNB5 markedly elevated the activity of Rac1 but not CDC42 and RhoA. Consistently, knockdown of RGS11 in CL1-5F4 cells significantly downregulated the Rac1 activity although the activity of RhoA was slightly increased (Supplementary Figure S6). This finding suggests that the signaling linkage in the Rac1–MAPK–FAK pathway is the major downstream signaling cascade initialed by RGS11–GNB5 and that is responsible for cell migration.

### Increased cell invasion by expression of urokinase plasminogen activator (uPA)

**Figure 6:**
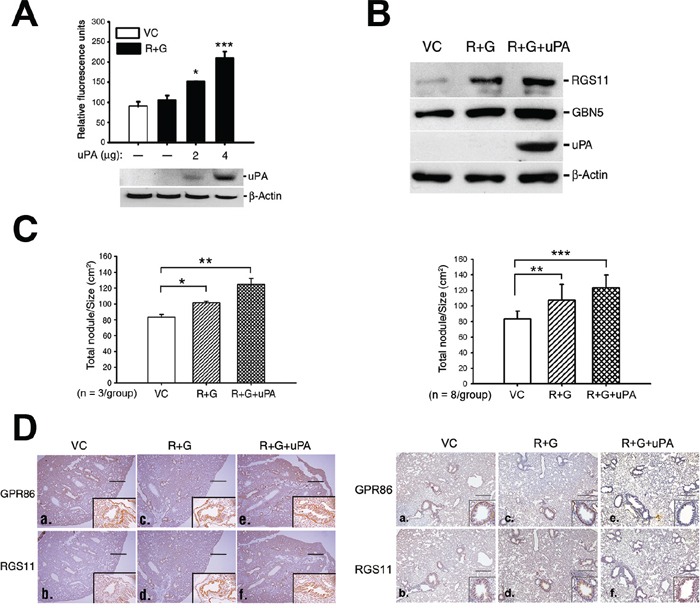
Augmentation of tissue invasion of the RGS11–GNB5 transfectants by coexpression of uPA **A.** Cell invasion assay. The RGS11–GNB5 CL1-0 transfectants (R+G) were transiently transfected with different doses of a plasmid encoding for His-tagged uPA, as indicated. After the 24 h transfection, the invasion capability was measured by chemoattraction of the cells in the 8 μm-pore size Transwell chamber coated with extracellular matrix for 24 h at 37°C. The cells that penetrated and migrated through the membranes were quantified fluorometrically using a spectrophotometer ELISA reader at 485 nm (emission)/538 nm (adsorption). The data are expressed as the mean ± SD of triplicate measurements. **B.** Western blot analysis. The R+G cells were transfected with 4 μg of the *uPA* plasmid, and the stable transfectants were selected with Zeocin™. The expression of uPA in the stable transfectants (R+G+uPA) was examined by Western blotting using antibody against uPA. Beta-Actin serves as a loading control. **C.** Qualification of metastatic colony formation in lungs. NOD-SCID mice (3 mice/group) were intravenously administrated with CL1-0 cells (1 × 10^6^/mouse) that coexpressed RGS11–GNB5 (R+G) or RGS11–GNB5–uPA (R+G+uPA) in the first experiment (*left*). An additional independent experiment was carried out in NOD-SCID mice (8 mice/group) as described above, shown in the right panel. Mice were sacrificed after 6 weeks. After immunohistochemical staining using antibody specific to GPR86 (a marker for CL1-0 cells) [33], lung nodules of each mouse were visualized and counted under light microscopy. The area of the lungs was imaged and calculated using ImageJ software. The results are expressed as the mean ± SD of the number of nodules/cm^2^ of lung. The data were analyzed using Student's *t* test. *, **, *** indicate *P* < 0.05, *P* < 0.01 and *P* < 0.001, respectively. **D.** IHC analysis. Transverse serial sections were obtained from mice that had been injected intravenously with the cells that stably coexpressed RGS11–GNB5 (R+G) or RGS11–GNB5–uPA (R+G+uPA), or their control cells (VC) and stained with antibodies specific to human RGS11 and GPR86. The first (3 mice/group) and second independent experiments (8 mice/group) were shown in the left and right panels, respectively. The cells displaying membrane-associated GPR86 and cytoplasmic RGS11 localized to the lining surface of alveolar tissues (insets in a–f). Magnification, 40× (a–f) as well as 400× (insets) in the first study and 200× (insets) in second study. Scale bar, 500 μm.

The *uPA* gene was introduced into the CL1-0 RGS11–GNB5 (R+G) transfectants to confer tissue-invading ability, and the metastatic colony formation in the lung was assessed. Cotransfection of the *uPA* gene increased the *in vitro* invasive capability of the cells in a dose-dependent manner (Figure 6A). After establishment of transfectants that stably expressed RGS11-GNB5 (R+G) or RGS11-GNB5-uPA (R+G+uPA), or their vector control (VC) (Figure 6B), the cells were administered intravenously into mice. Three mice per group were used in the first experiment. The mice were sacrificed, and the number of metastatic nodules and the total area of lung lobes affected in each mouse were counted and quantified using ImageJ software. The cells that stably expressed RGS11–GNB5 (R+G) showed only slightly more nodules per unit volume (N/cm^2^) compared with control mice. Notably, additional expression of uPA in the cells (R+G+uPA) conferred a significant increase in tissue invasion (Figure 6C and 6D; *left*). To further confirm the data, an additional independent experiment using 8 mice per group was carried out. The result shows that CL1-0 transfectants stably expressing RGS11–GNB5 (R+G) in the presence or absence of additional expression of uPA all displayed a significant increase in formation of metastatic colony in lungs (Figure 6C and 6D; *right*), in particular, the group with additional expression of uPA. These findings suggest that the pathological role of RGS11–GNB5 in tumor metastasis mainly promotes the migration of lung cancer cells.

## DISCUSSION

Tumor metastasis is the leading cause of cancer-related death of lung cancer patients. Lymph node (LN) metastasis is one possible mechanism by which primary tumors spread to distal organs such as bones. In the present study, we used subtractive hybridization to identify the differentially expressed genes involved in the metastatic process. We found that RGS11 was highly expressed in LN- and bone-metastatic tumors as well as advanced primary tumors in patients with lung adenocarcinoma (Figure 1). Its overexpression was significantly associated with increments in the primary tumor status, nodal metastasis, and disease stage, and with poorer DSS and DFMS of the patients (Table 1–3; Figure 2). Similarly, RGS4 has been reported to be a key driver of glioblastoma invasiveness [6]. Overexpression of RGS5 has been detected in hepatocellular carcinoma and is associated with cancer recurrence, venous infiltration, and poorer survival [18]. Increased expression of RGS17 in prostate and lung cancers has been shown to support cancer progression [7]. Additionally, *RGS11* has also been validated as one of the genes upregulated in colorectal cancer and implicated in the acquisition of oxaliplatin resistance [16]. Thus, although RGS11 was originally characterized as the key GAP that controls the onset of the light response in ON bipolar cells [19, 20], follicular maturation [21] and neural progenitor cell differentiation [22], its overexpression also seems to be strongly associated with tumor metastasis and poor clinical outcomes in patients with lung adenocarcinoma.

The present study pinpoints the pathological role of RGS11 in cell migration. Cell migration is a complex and coordinated cellular process that is essential for the local and distal spread of tumor cells. Remodeling of the actin cytoskeleton is required for altered motile phenotypes and often associated with cancer progression. Extracellular stimuli such as hormones and chemokines can initiate intracellular signaling cascades by linking their receptors with selective receptor-associated GPCRs, activating small GTP-bound proteins and subsequently re-organizing cytoskeleton leading to cell shape changes. In this biological process, RGS proteins may carry out their GAP function by modulating the Gα activity of GPCRs. In this study, the expression status of RGS11 was associated with cell mobility (Figure 3A-3C). The expression of RGS11 was higher in fast-migrating CL1-5F4 cells than in slow-migrating CL1-0 cells (Figure 3A). Experiments involving manipulation of RGS11 expression showed clearly the role of RGS11 in the promotion of cell migration, but not cell invasion (Figure 3D-3H) or cell proliferation (Supplementary Figure S5). The actions driven by RGS11 occurred through upregulation of Rac1 activity and followed by activation of the Raf–ERK–FAK signaling linkage (Figures 4 and 5). The same biological readout with different mechanism is also found in other RGS protein. RGS4-mediated glioma cell invasion is signaled by mTOR activation [6]. Tumor metastasis mediated by RGS5 occurs through induction of the epithelial–mesenchymal transition in liver cancer cells [18]. Thus, in contrast to studies showing a general role of RGS proteins in the negative regulation of migration of normal lymphoid cells [23, 24], our data lead us to speculate that the role of RGS proteins in the migration of cancer cells is more complicated than the known functions of RGS in physiological conditions, in particular for those RGS proteins upregulated in cancers.

Some RGS proteins have distinct functions in cell migration that depend on the cell and tissue types. A general property of GPCR-initiated signaling is that prolonged stimulation decreases responsiveness (desensitization). Recruitment of an RGS protein to the vicinity of the ligand-activated receptor–G protein complex is thought to provide an additional mechanism to regulate the signaling. Overexpression of RGS3 and RGS4 leads to the blockade of Gα-dependent chemotaxis in lymphoid cells [24], whereas increasing expression of either of these RGS proteins increases cell adhesion and chemotaxis in glioma cells [25]. On the other hand, in breast cancer cells, RGS4 suppresses Ras-related C3 botulinum toxin substrate 1-dependent lamellipodia formation [26]. However, gene silencing of *RGS4* or pharmacological inhibition of its protein reduced cell mobility and invasiveness in glioma cells and in an intracerebral mouse model, respectively [6]. In the abovementioned studies, RGS proteins did not always act as a negative regulator of their biological actions, in particular, cell mobility. The expression of numerous RGS proteins have been reported to be altered in a variety of cancers [2], which suggests that the actions may depend on the cell contexts and tissue types and may involve activation or inactivation of selective subsets of signaling pathways. In the present study, RGS11 was dominantly expressed in brain neurons and retinal bipolar cells but was weakly expressed in other tissues in physiological conditions. However, overexpression of RGS11 was found in samples of lung adenocarcinoma. Cotransfection of *RGS11* with additional protease genes such as *uPA* in CL1-0 cells significantly elevated the capability of cell and tissue invasion (Figure 6), confirming its newly-identified role in cell migration and participation in tumor metastasis.

Despite the compelling evidence that many RGS proteins, including those of the R7 RGS family, are upregulated and associated with disease progression, the mechanisms underlying GPCR-mediated signalings driven by these proteins are unclear. RGS6, a member of the R7 RGS subfamily that shares an overall structural organization with RGS11 and is required for the activity of doxorubicin to activate ATM–p53-induced apoptosis [27]. Exogenous expression of an RGS6 mutant that does not interact with the GTPase activity of the Gα subunit was as capable as its wild-type counterpart in promoting p53 activation. This unexpected result indicates that the action of RGS6 was independent of its ability to interact with G protein and suggests that there are uncharacterized signaling activities mediated by the Gα-independent regulation for some RGS proteins, especially in cancer cells. RGS3 and RGS4, which share a similar function with RGS11 in modulating Gα_i/o_ activity [28, 29] are overexpressed in faster migrating glioma cells, which results in greater cell adhesion and chemotaxis [25]. A similar finding was also seen in our preliminary study. Overexpression of RGS11–GNB5 markedly increased the binding preference of CL1-0 cells to plates coated with extracellular matrix fibronectin and collagen I (data not shown) and further supported its role in cell migration. However, the mechanism(s) through which G protein-dependent and -independent signaling activities are mediated remain unclear and await further study.

The present study is the first to identify a role of RGS11 in the promotion of cell migration of cancer cells and show its overexpression highly associated with poorer prognosis of patients with lung adenocarcinoma. In order to verify RGS11 as a potential diagnostic and/or prognostic biomarker for lung adenocarcinoma, both specificity and sensitivity of RGS11, other R7 RGS, and their associated proteins are being estimated in a larger cohort of patients with adenocarcinoma and other subtypes of lung cancer currently. In addition, a physic association of RGS11 with GNB5 is particularly highlighted to be a key factor for promotion of cell migration in present study. Hence, we believe that understanding of RGS11 may promise not only to facilitate new drug development by targeting its protein interaction with GNB5, but also to provide new insights into its downstream signalings that may be relevant to understanding the causes and treatments of tumors that overexpress RGS11.

## MATERIALS AND METHODS

### Patients

Formalin-fixed, paraffin-embedded tissues and clinical information were obtained from 12 lung cancer patients with paired primary and bone metastatic adenocarcinoma, as well as from 91 patients with lung adenocarcinoma. The tissues and clinical information were obtained between 1999 and 2002 from the Tissue Bank of the Chi-Mei Medical Center. The study was approved by the Institutional Review Boards of the center (IRB100-05-003). Histological types and tumor grading were determined according to the 2000 WHO classification. Disease staging was scored by tumor size and node metastasis based on the 7^th^ edition of AJCC system.

### Cells and cell culture

NCI-H23, the highly invasive CL1-5F4 and the poorly invasive CL1-0 cells were cultured in RPMI 1640 medium supplemented with 10% fetal bovine serum (FBS), penicillin (100 units/mL), and streptomycin (100 mg/mL) (Invitrogen, Carlsbad, CA). NHBE cells were maintained in bronchial epithelial cell basal medium (ScienCell, Carlsbad, CA) plus 20% FBS. The information regarding to generation of CL1-0 transfectants stably expressing RGS11/GNB5 (R+G) or RGS11/GNB5/uPA (R+G+PA) as well as CL1-5F4 transfectants with attenuated expression of RGS11 (sh-RGS11) is given in the Supplementary Materials and Methods section.

### Western blot analysis

The cells were lysed in cell lysis buffer [30], and the protein concentration was measured using the BCA assay (Thermo Scientific, Rockford, IL). Western blot analysis was performed for protein expression using antibodies against RGS11, GNB5, phospho (p)-FAK (Ser910), or RGS9 (Abcam, Cambridge, UK); antibodies against p-c-Raf (Ser338), FAK, ERK, or p-ERK (Thr202/Tyr204) (Cell Signaling Technology, Beverly, MA); or antibodies against c-Raf, RGS6, or RGS7 (GeneTex, San Antonio, TX).

### Transwell cell migration and invasion assays

The capacities for cell migration and invasion were determined using *in vitro* Boyden-chamber Transwell^®^ analysis and a fluorometric detection system (Chemicon/Millipore, Temecula, CA). The Transwell inserts with an 8 μm-pore size polycarbonate membrane that were coated or uncoated with tumor-associated matrix were used for the cell invasion and migration assays, respectively. NHBE cells and tumor cells (5 × 10^5^/well) were starved in 0.1% FBS-containing appropriate culture medium at 37°C for 2 h and then seeded into the upper chamber of the Transwell system. The migration or invasion through the membrane was driven by 10% FBS-containing medium in the lower chamber for 6 h (migration assay) or 24 h (invasion assay). The cells moving through the membranes were quantified fluorometrically according to the manufacturer's instructions. The fluorometric signals were measured using a Spectramax ELISA reader (Molecular Devices, Sunnyvale, CA) at 485/538 nm.

### Reverse transcription-polymerase chain reaction (RT-PCR)

Total RNA of cells was purified using the RN easy Mini kit (Qiagen, Hilden, Germany) and reversely transcribed using Superscript III reverse transcriptase (Invitrogen). The gene amplifications were performed as described in Supplementary Materials and Methods.

### Measurement of the activity of the Rho family

CL1-0 transfectants stably expressing RGS11-GNB5 (R+G) or their corresponding vectors (VC) were grown to 70–80% confluence and lysed. Active GTP-bound RhoA, Rac1, or CDC42 in the lysates (100 μg/sample) were isolated by binding to their corresponding associated proteins, Rhotekin–RBD and PAK–PBD, and detected with antibodies specific to RhoA, Rac1, or CDC42 in an absorbance-based G-LISA^®^ system (Cytoskeleton, Denver, CO) at 490 nm. To detect directly the protein–protein interactions, a pull-down assay was performed using the Rho Family Activation Kit (Millipore). After cells lysis, the active forms of RhoA, Rac1, and CDC42 were pulled down with their corresponding glutathione-S-transferase (GST)-tagged associated proteins conjugated with glutathione–agarose beads, according to the manufacturer's instructions. The GTP-bound Rho family members were detected by their specific antibodies and visualized using the SuperSignal system (Thermo Scientific).

### Lung metastatic colony-formation assay

Non-obese diabetic–severe combined immunodeficiency (NOD-SCID) mice (6–8 weeks old) were obtained from the National Laboratory Animal Center, Taiwan. The mice (3/group in the first study and 8/group in an additional independent study) were intravenously administered with CL1-0 cells (1 × 10^6^/mouse) that coexpressed RGS11–GNB5 or RGS11–GNB5–uPA, or their control cells. After 6 weeks, the mice were sacrificed. The lung tissues were fixed and embedded in paraffin. Serial tissue sections were immunostained with antibodies to human RGS11 or GPR86 (a marker for CL1-0; Abcam) [33] (1:40 and 1:500 dilutions, respectively). The numbers of tumor nodules in the lungs of each mouse were visualized under light microscopy and were counted by two experienced pathologists. The whole area of the lungs was imaged and calculated using the ImageJ software. The results are expressed as the mean ± SD of the number of nodules/cm^2^ of lung. Student's *t* test was used to identify significant differences.

### Immunoprecipitation and immunoblots

CL1-0 cells stably expressing RGS11 and GNB5 (R+G) and their mock-transfected control cells were grown to 80-90% confluence in complete medium, as described above. The cells washed twice in PBS and fixed with 4% paraformaldehyde for 20 min. After washed with PBS twice, cells were lysed in radioimmunoprecipitation assay buffer (RIPA) with protease inhibitor cocktail (Roche Diagnostics, Indianapolis, IN) and orthovanadate (Sigma-Aldrich, Saint Louis, MO). Lysates were clarified with centrifugation at 1,500 × g for 10 min at 4°C to remove cell debris, and the supernatant was pre-cleared with 250 μL of Protein A/G Sepharose beads (Thermo Scientific). The protein concentration of the supernatant was quantified with bicinchoninic acid assay (BCA; Thermo Scientific). The total protein (500 μg) was incubated with purified home-made murine antibody specific to RGS11 (20 μg/reaction) for 2 h, and followed by addition of 50 μL of Protein A/G Sepharose beads with gently rocking at 4°C overnight. The antibody/protein/Sepharose complex was washed three times with RIPA buffer. Proteins were denatured in boiling Laemmli sample buffer, resolved by SDS-PAGE, and Western blotted with antibody against RGS11, GNB5 (Abcam) or RhoA/Rac1/CDC42 in the G-LISA systems (Cytoskeleton).

### Immunohistochemical (IHC) study and statistical analysis

The procedures for immunohistochemistry were performed as previously described [30, 31]. The information is given in the Supplementary Materials and Methods section.

## SUPPLEMENTARY MATERIALS FIGURES



## References

[1] Gutkind JS (1998). The pathways connecting G protein-coupled receptors to the nucleus through divergent mitogen-activated protein kinase cascades. J Bio Chem.

[2] Hurst JH, Hooks SB (2009). Regulator of G-protein signaling (RGS) proteins in cancer biology. Biochem pharmacol.

[3] Rozengurt E (2007). Mitogenic signaling pathways induced by G protein-coupled receptors. J Cell Physiol.

[4] Berman DM, Kozasa T, Gilman AG (1996). The GTPase-activating protein RGS4 stabilizes the transition state for nucleotide hydrolysis. J Biol Chem.

[5] Dorsam RT, Gutkind JS (2007). G-protein-coupled receptors and cancer. Nature reviews Cancer.

[6] Weiler M, Pfenning PN, Thiepold AL, Blaes J, Jestaedt L, Gronych J, Dittmann LM, Berger B, Jugold M, Kosch M, Combs SE, von Deimling A, Weller M, Bendszus M, Platten M, Wick W (2013). Suppression of proinvasive RGS4 by mTOR inhibition optimizes glioma treatment. Oncogene.

[7] Bodle CR, Mackie DI, Roman DL (2013). RGS17: an emerging therapeutic target for lung and prostate cancers. Future Med Chem.

[8] Cao X, Qin J, Xie Y, Khan O, Dowd F, Scofield M, Lin MF, Tu Y (2006). Regulator of G-protein signaling 2 (RGS2) inhibits androgen-independent activation of androgen receptor in prostate cancer cells. Oncogene.

[9] Schwable J, Choudhary C, Thiede C, Tickenbrock L, Sargin B, Steur C, Rehage M, Rudat A, Brandts C, Berdel WE, Muller-Tidow C, Serve H (2005). RGS2 is an important target gene of Flt3-ITD mutations in AML and functions in myeloid differentiation and leukemic transformation. Blood.

[10] Chen X, Higgins J, Cheung ST, Li R, Mason V, Montgomery K, Fan ST, van de Rijn M, So S (2004). Novel endothelial cell markers in hepatocellular carcinoma. Mod Pathol.

[11] Boss CN, Grunebach F, Brauer K, Hantschel M, Mirakaj V, Weinschenk T, Stevanovic S, Rammensee HG, Brossart P (2007). Identification and characterization of T-cell epitopes deduced from RGS5, a novel broadly expressed tumor antigen. Clin Cancer Res.

[12] Sandiford SL, Slepak VZ (2009). The Gbeta5-RGS7 complex selectively inhibits muscarinic M3 receptor signaling via the interaction between the third intracellular loop of the receptor and the DEP domain of RGS7. Biochemistry.

[13] Jayaraman M, Zhou H, Jia L, Cain MD, Blumer KJ (2009). R9AP and R7BP: traffic cops for the RGS7 family in phototransduction and neuronal GPCR signaling. Trends Pharmacol Sci.

[14] Anderson GR, Posokhova E, Martemyanov KA (2009). The R7 RGS protein family: multi-subunit regulators of neuronal G protein signaling. Cell Biochem Biophys.

[15] Dai J, Gu J, Lu C, Lin J, Stewart D, Chang D, Roth JA, Wu X (2011). Genetic variations in the regulator of G-protein signaling genes are associated with survival in late-stage non-small cell lung cancer. PloS One.

[16] Martinez-Cardus A, Martinez-Balibrea E, Bandres E, Malumbres R, Gines A, Manzano JL, Taron M, Garcia-Foncillas J, Abad A (2009). Pharmacogenomic approach for the identification of novel determinants of acquired resistance to oxaliplatin in colorectal cancer. Mol Cancer Ther.

[17] Zheng Y, Xia Y, Hawke D, Halle M, Tremblay ML, Gao X, Zhou XZ, Aldape K, Cobb MH, Xie K, He J, Lu Z (2009). FAK phosphorylation by ERK primes ras-induced tyrosine dephosphorylation of FAK mediated by PIN1 and PTP-PEST. Mol Cell.

[18] Hu M, Chen X, Zhang J, Wang D, Fang X, Wang X, Wang G, Chen G, Jiang X, Xia H, Wang Y (2013). Over-expression of regulator of G protein signaling 5 promotes tumor metastasis by inducing epithelial-mesenchymal transition in hepatocellular carcinoma cells. J Surg Oncol.

[19] Cao Y, Pahlberg J, Sarria I, Kamasawa N, Sampath AP, Martemyanov KA (2012). Regulators of G protein signaling RGS7 and RGS11 determine the onset of the light response in ON bipolar neurons. Proc Natl Acad Sci USA.

[20] Chen FS, Shim H, Morhardt D, Dallman R, Krahn E, McWhinney L, Rao A, Gold SJ, Chen CK (2010). Functional redundancy of R7 RGS proteins in ON-bipolar cell dendrites. Invest Ophthal Vis Sci.

[21] Barzilay E, Yung Y, Shapira L, Haas J, Ophir L, Yerushalmi GM, Maman E, Hourvitz A (2014). Differential expression of poliovirus receptor, regulator of G-protein signaling 11 and erythrocyte protein band 4.1-like 3 in human granulosa cells during follicular growth and maturation. Gynecol Endocrinol.

[22] Tuggle K, Ali MW, Salazar H, Hooks SB (2014). Regulator of G Protein Signaling Transcript Expression in Human Neural Progenitor Differentiation: R7 Subfamily Regulation by DNA Methylation. Neurosignals.

[23] Moratz C, Harrison K, Kehrl JH (2004). Role of RGS proteins in regulating the migration of B lymphocytes. Arch Immunol Ther Exp.

[24] Bowman EP, Campbell JJ, Druey KM, Scheschonka A, Kehrl JH, Butcher EC (1998). Regulation of chemotactic and proadhesive responses to chemoattractant receptors by RGS (regulator of G-protein signaling) family members. J Biol Chem.

[25] Tatenhorst L, Senner V, Puttmann S, Paulus W (2004). Regulators of G-protein signaling 3 and 4 (RGS3, RGS4) are associated with glioma cell motility. J Neuropathol Exp Neurol.

[26] Xie Y, Wolff DW, Wei T, Wang B, Deng C, Kirui JK, Jiang H, Qin J, Abel PW, Tu Y (2009). Breast cancer migration and invasion depend on proteasome degradation of regulator of G-protein signaling 4. Cancer Res.

[27] Huang J, Yang J, Maity B, Mayuzumi D, Fisher RA (2011). Regulator of G protein signaling 6 mediates doxorubicin-induced ATM and p53 activation by a reactive oxygen species-dependent mechanism. Cancer Res.

[28] Hooks SB, Waldo GL, Corbitt J, Bodor ET, Krumins AM, Harden TK (2003). RGS6, RGS7, RGS9, and RGS11 stimulate GTPase activity of Gi family G-proteins with differential selectivity and maximal activity. J Biol Chem.

[29] Moratz C, Kehrl JH (2004). In vitro and in vivo assays of B-lymphocyte migration. Methods Mol Biol.

[30] Chang GC, Liu KJ, Hsieh CL, Hu TS, Charoenfuprasert S, Liu HK, Luh KT, Hsu LH, Wu CW, Ting CC, Chen CY, Chen KC, Yang TY, Chou TY, Wang WH, Whang-Peng J (2006). Identification of alpha-enolase as an autoantigen in lung cancer: Its overexpression is associated with clinical outcomes. Clin Cancer Res.

[31] Huang HY, Kang HY, Li CF, Eng HL, Chou SC, Lin CN, Hsiung CY (2006). Skp2 overexpression is highly representative of intrinsic biological aggressiveness and independently associated with poor prognosis in primary localized myxofibrosarcomas. Clin Cancer Res.

[32] GNB5 interacting proteins in the protein-protein interaction dataset: http://amp.pharm.mssm.edu/Harmonizome/gene_set/GNB5/Pathway+Common+Protein-Protein+Interactions

[33] GPR86 as a marker for CL1-0 xenografted tumor in mice: http://www.genetex.com/GPR86-antibody-N3C2-Internal-GTX100380.html

