# Supplementary material for: Overexpression of regulator of G protein signaling 11 promotes cell migration and associates with advanced stages and aggressiveness of lung adenocarcinoma
Source: Oncotarget. 2016 Apr 20;7(21):31122–36. doi: 10.18632/oncotarget.8860 (PMC5058744; doi:10.18632/oncotarget.8860)
Supplement: Supplementary file 1 [file oncotarget-07-31122-s001.pdf]

# SUPPLEMENTARY DATA

## Generation of stable transfectants

To generate cells that stably overexpressed RGS11, CL1-0 cells were transfected with pcDNA–RGS11, pcDNA–GNB5, or both plasmids using Arrest-In (Open Biosystems, Huntsville, AL). The cells were then treated with appropriate antibiotics (400 mg/mL of G418 or 200 µg/mL hygromycin B; Thermo Scientific, Rockford, IL) and subjected to limiting dilution. The stable transfectants with coexpression of RGS11 and GNB5 were transfected with the pcDNA-His-uPA plasmid. The resultant cells were selected in medium containing 150 µg/mL Zeocin (Thermo Scientific) to generate stable cells expressing His-tagged uPA. CL1-5F4 cells were transfected with the pLKO–sh-RGS11 plasmid, which transcribes RGS11-specific shRNA (Academia Sinica, Taipei, Taiwan) to generate transfectants with attenuated expression of RGS11, using Lipofectamine 2000 (Invitrogen). The cells were then treated with 2.5 µg/mL of puromycin (Thermo Scientific) and subjected to limiting dilution. Stable clones with high or knocked-down expression of RGS11 were determined by Western blot analysis. Simultaneously, their corresponding stable control cells were also established by transfecting either the pcDNA3.1 or pLKO.1 vector. FAK was attenuated in the RGS11–GNB5-transfected CL1-0 (R+G) cells by transfection with its specific sh-RNA construct (Academia Sinica, Taipei, Taiwan).

## Subtraction hybridization by phenol emulsion reassociation

To identify metastasis-related genes, two pairs of fresh primary lung adenocarcinoma tissues and lymph-node metastatic counterparts were collected under approval of the Institutional Review Boards of the Chi-Mei Medical Center (IRB100-05-003). Genes differentially expressed between primary and lymph-node metastatic lung adenocarcinoma tissues were highly enriched by subtraction hybridization by phenol emulsion reassociation technique (PERT) [1, 2], with some modifications. In brief, total RNA of the primary and metastatic tissues was purified using the RNeasy Mini kit (Qiagen, Hilden, Germany) and reversely transcribed using Superscript III reverse transcriptase (Invitrogen) and oligo-$(dT)_{18}$ as primer to generate first-stranded cDNA. Synthesis of double-stranded cDNA for subtractive hybridization was performed using the Subtractor kit purchased from Invitrogen (Carlsbad, CA). The cDNA pool from the primary tissues was biotinylated using biotinylated nested primers (forward: 5’-biotin-GGACACTGACATGGACTGAAGGAGTA-3’; backward: 5’-biotin-CGCT-ACGTAACGGCATGACAGTG-3’) by PCR and acted as driver cDNA in this case; on the other hand, the cDNA pool from the metastatic tissues was ligated with adaptors (forward: 5’-AATGCGGCCGCACTATAGCATGGACTG AAGGAGTA-3’; backward: 5’-CATTTAGGTGACACTA GTAACGGCATGACAGTG-3’) and as tester cDNA. The cDNA pools were PCR amplified essentially as described by Wang and Brown [3]. Removal of unligated linkers and free nucleotides in PCR reactions were carried out using the GeneClean III system (Q·Biogene, Carlsbad, CA), as instructed in the manufacturer’s manual. Subsequently, the biotinylated or adaptor-ligated cDNA pool was collected into water. The adaptor-ligated tester cDNA pool was mixed with 10-fold excess amount of biotinylated driver cDNA pool in a solution containing 40 mM Tris-HCl, pH8.0 / 4 mM EDTA, and denatured at 95°C for 5 min. Hybridization was carried out by PERT in a 500 µL-solution containing 2 M NaSCN / 8% phenol at room temperature (RT) for 24 h, as described by Kohne et al. [4], with continuous agitation on Vortex Mixer (Pittsburgh, PA). After hybridization, the solution was extracted with equal volume of chloroform/isoamyl alcohol (24:1). The aqueous layer was collected, added with 1 µL of 10 mg/mL yeast tRNA / 50 µL of 8 M ammonium acetate / 300 µL of ice-cold absolute ethanol per 100 µL of the aqueous layer, and mixed well. The cDNA mixture was precipitated at –70°C for 30 min, centrifuged at 16,000 ×g, 4°C for 10 min, and washed with ice-cold 70% ethanol three times. After air dry, the cDNA pellet was resuspended in 10 mM HEPES/EDTA buffer containing 10 mg/mL streptavidin, mixed, and incubated for 10 min at RT. The biotinylated cDNA fragments were removed by repeated the phenol/chloroform extraction procedure for three times. The adaptor-ligated resultant pool was re-precipitated as described above, washed with ice-cold 70% ethanol, and resuspended in water.

Construction of subtractive library and clonal analysis by PCR was performed as described by Zeng, et al. [2], with some modifications. After subtraction, the subtracted PCR products were amplified by random primers, cloned into TA vector and transformed into competent INVaF' cells (TA cloning kit, Invitrogen). For clonal analyses, white colonies were picked, and inserted genes were identified by direct sequencing.

## Reverse transcription-polymerase chain reaction (RT-PCR)

To determine the gene expression profile of R7 RGS subfamily members, total RNAs of CL1-0 and CL1-5F4 cells were purified using the RNeasy Mini kit (Qiagen, Hilden, Germany) and reversely transcribed by the Superscript III reverse transcriptase (Invitrogen, Carlsbad, CA). The gene amplifications were performed

in PCR reactions containing the Ampliqon III DNA polymerase (Ampliqon, Copenhagen, Denmark) and gene-specific primers, as listed in the followings, for 30 cycles. The primers for RGS6 (forward: TGCCAACCTGGCTGTGAGGTAATG; backward: CCTAATGCC-CGCGATGGCACT), RGS7 (forward: AGAAGATCCAGTGGAGGCGCTC; backward: GGCCTGTGCACGTCCCAGAAC), RGS9 (forward: ACGCCATCTA-TCTGGCCAAGCG; backward: TGACAGCAACGACTGTTTGTTTCTGG), RGS11 (forward: ACATGTACAAGGCCCTCCTG; backward: GCTCACGAAGGAAGAC-TTGG), GNB5 (forward: ACCATGGCAACCGAGGGGCT; backward: TTAGGCC-CAGACTCTGAG), and β-actin (forward: GGTCACCCACACTGTGCCCATCTA; backward: GAAGCATTGCGGTGGACGATGGAG). The resultant products were resolved in 1% ethidium bromide-containing agarose gel (Supplementary Figure 3).

## Cell growth

Stable transfectants and their corresponding control cells ($5 \times 10^3$ cells per well) were seeded into 96-well plates and incubated for 24, 48, 72 or 96 h (Supplementary Figure 5). Cell proliferation was determined by incubating cells in 200 µL of fresh appropriate medium containing 1 mg/mL 3-(4,5-dimethylthiazol-2-yl)-2,5-diphenyltetrazolium bromide (MTT) (Sigma-Aldrich, Saint Louis, MO) for 4 h at 37°C. After removal of the MTT solution, the resulting formazan crystals were dissolved completely in an ethanol/dimethyl sulfoxide mixture (1:1), and the plates were read using SpectraMax M5 ELISA reader (Molecular Devices, Sunnyvale, CA). Data are expressed as the mean ± SD of triplicates.

## Immunohistochemistry (IHC) staining and statistical analysis

IHC technique was performed as previously described [5, 6]. In brief, sections were cut onto adhesively coated glass slides at 5-µm thickness, dewaxed, rehydrated with phosphate-buffered saline, and incubated with RGS11 antibody (1:40 dilution; Abcam). Immune complexes were detected using the ChemMate DAKO EnVision kit (K5001; DAKO, Carpinteria, CA), and the slides were counterstained with hematoxylin. Negative controls were made by incubating the corresponding serial tissue sections with the antigen-absorbed primary antibody. The RGS11 expression status was determined by two experienced pathologists. The percentage of tumor cells with moderate or strong cytoplasmic immunoreactivity was recorded. The extent of immunoreactivity was scored as negative (0+, <5%), weak (1+, ≥5% but <25%), moderate (2+, ≥25% but <50%), strong (3+, ≥50% but <75%), and intense (4+, ≥75%). Tumors displaying 0 to 2+ immunostaining were classified as low expression, while those displaying 3 to 4+ immunostaining were classified as high expression.

Statistical analyses were performed using the SPSS 14 software package. The associations between various clinicopathological parameters and RGS11 expression status were evaluated using the chi-square test as appropriate. The end points analyzed were DSS and DMFS, which were calculated from the date of surgery until cancer death or development of distal metastasis, respectively, or the last follow-up appointment. The median period of follow-up was 21.3 months (range, 1–86.7). Univariate survival analysis was performed using Kaplan–Meier plots and the survival variables were compared using the log-rank test. A multivariate model using Cox proportional-hazards regression included the parameters with a univariate $P$-value < 0.05.

## REFERENCES


1. Barr FG, Emanuel BS. Application of a subtraction hybridization technique involving photoactivatable biotin and organic extraction to solution hybridization analysis of genomic DNA. Anal Biochem. 1990; 186:369-373.
2. Zeng J, Gorski RA, Hamer D. Differential cDNA cloning by enzymatic degrading subtraction (EDS). Nucleic Acids Res. 1994; 22:4381-4385.
3. Wang Z, Brown DD. A gene expression screen. Proc Natl Acad Sci U S A. 1991; 88:11505-11509.
4. Kohne DE, Levison SA, Byers MJ. Room temperature method for increasing the rate of DNA reassociation by many thousandfold: the phenol emulsion reassociation technique. Biochemistry. 1977; 16:5329-5341.
5. Chang GC, Liu KJ, Hsieh CL, Hu TS, Charoenfuprasert S, Liu HK, Luh KT, Hsu LH, Wu CW, Ting CC, Chen CY, Chen KC, Yang TY, Chou TY, Wang WH, Whang-Peng J, et al. Identification of alpha-enolase as an autoantigen in lung cancer: Its overexpression is associated with clinical outcomes. Clin Cancer Res. 2006; 12:5746-5754.
6. Huang HY, Kang HY, Li CF, Eng HL, Chou SC, Lin CN, Hsiung CY. Skp2 overexpression is highly representative of intrinsic biological aggressiveness and independently associated with poor prognosis in primary localized myxofibrosarcomas. Clin Cancer Res. 2006; 12:487-498.

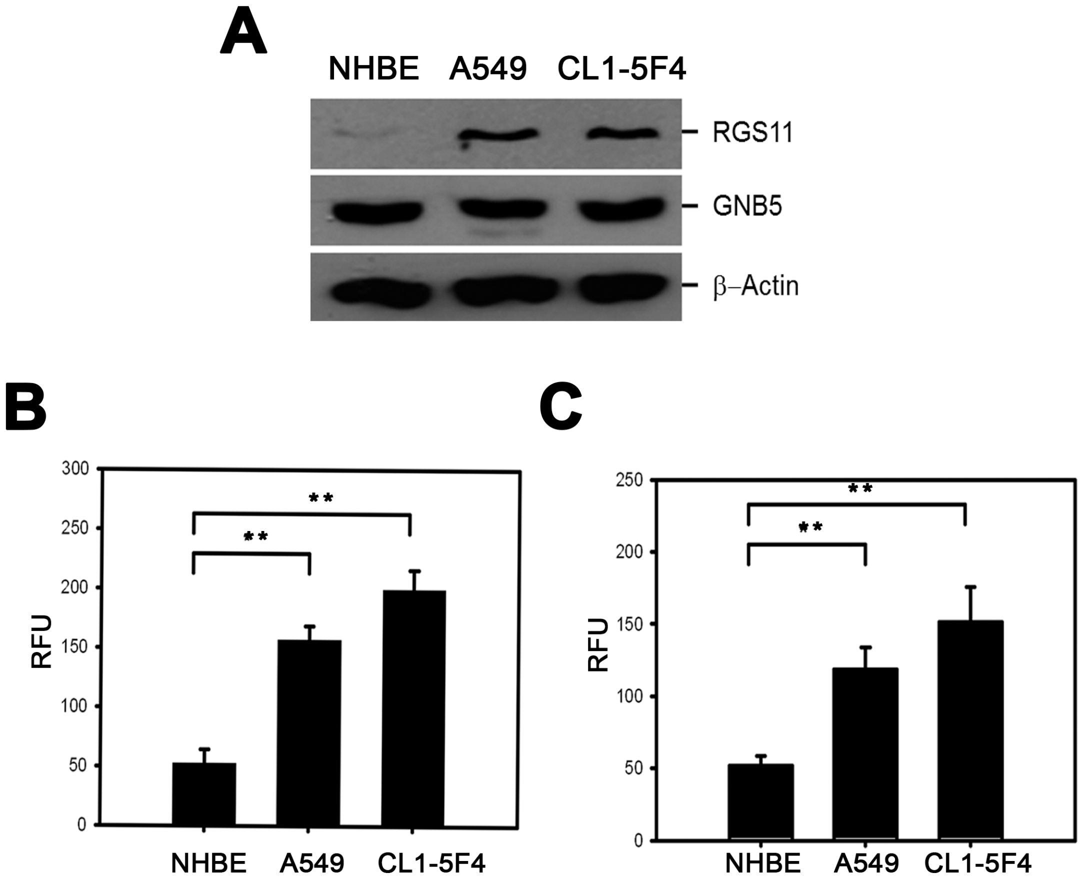


**Supplementary Figure S1: Correlation of RGS11 expression status with cell migration and invasion. A.** Western blot analysis. The cell lysates of NHBE, A549, and CL1-5F4 cells were resolved by SDS-PAGE, and the protein bands were immunoblotted with antibody specific to RGS11 or GNB5, as indicated on the right. NHBE cells and β-Actin serve as a normal lung cell control and a protein loading control, respectively. **B-C.** Transwell cell migration and invasion assays, respectively. Cells ($5 \times 10^5$ / reaction) chemo-attracted in Boyden-chamber Transwell assays were performed as described in the previous "Materials and Methods" section. The cells that moved through the membranes were quantified using a fluorometric detection system according to the manufacturer's instruction. The experiments were performed in triplicate. Data are expressed as the mean ± SD. ** indicates $P < 0.01$.

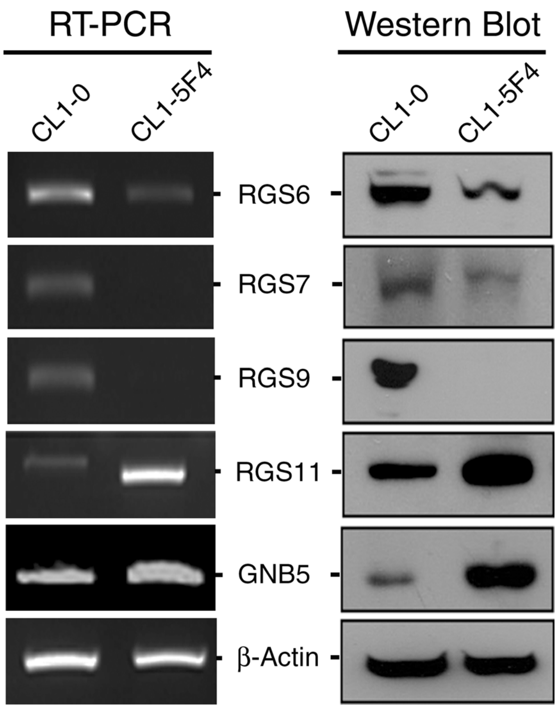


**Supplementary Figure S2: Expression profile of R7 RGS subfamily members.** The differential expression levels of RGS6, RGS7, RGS9, RGS11, as well as their associated protein, GNB5, between highly-invasive CL1-5F4 cells and their counterpart cells, CL1-0, were examined closely by RT-PCR (*left*) and Western blot (*right*) analyses. The total RNAs were extracted and reversely transcribed into cDNAs. The gene abundance of R7 RGS subfamily members was determined by PCR using gene-specific primers, and the resultant products were revealed on 1% ethidium bromide-containing agarose gel. Additionally, 50 µg lysates were resolved in 10% SDS-PAGE and immunoblotted with antibody specific to RGS6, RGS7, RGS9, RGS11 and GNB5, as indicated. Beta-Actin acts as a loading control.

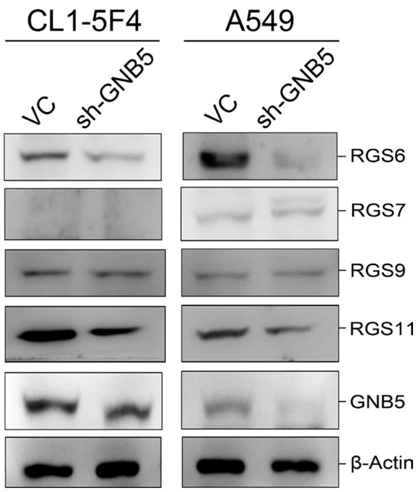


**Supplementary Figure S3: Knockdown of GNB5 destabilized expression of R7 RGS subfamily members.** CL1-5F4 and A549 cells stably transfected with a plasmid to transcribe small hairpin interfering RNA specific to endogenous GNB5 (sh-GNB5) were lysed. Western blot analysis of the R7 RGS members and GNB5 expression was carried out by immunoblotting with antibody against RGS6, RGS7, RGS9, RGS11 as well as GNB5, as indicated. Beta-Actin acts as a loading control.

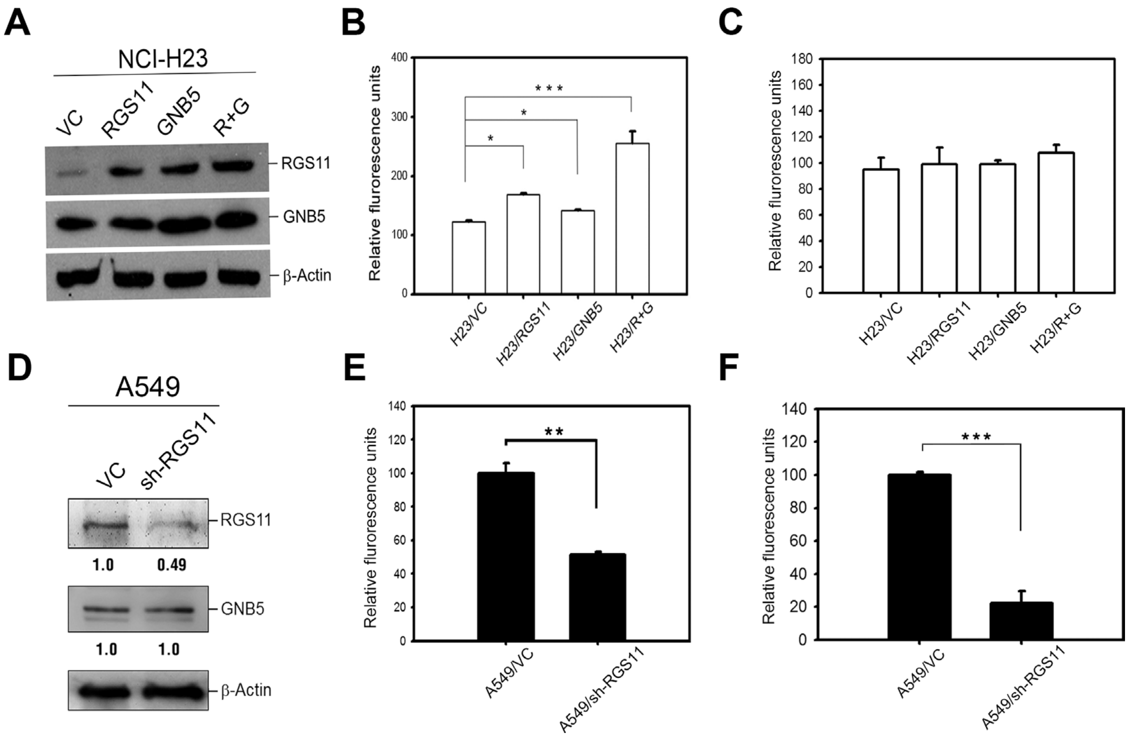


**Supplementary Figure S4: Modulation of RGS11 gene expression affected the migratory and invasive capabilities. A-C.** Increased RGS11 and GNB5 expression in NCI-H23. The cells were transiently transfected with a plasmid encoding for RGS11, GNB5, or combination of RGS11 and GNB5 (R+G). The expression levels of RGS11 and GNB5 were examined by Western blotting (A). Cells transfected with their corresponding empty vectors serve as control (VC). Cell migration (B) and invasion (C) capabilities of the transfectants was quantitatively determined by the Boyden chamber Transwell assays, as described previously. **D-F.** Decreased RGS11 expression in A549. Endogenous RGS11 of A549 cells were attenuated by transfecting a plasmid to transcribe RGS11-specific small hairpin interfering RNA (sh-RGS11). The expression level of RGS11 and GNB5 in the cells was determined by Western blot analysis (D). Cell migration (E) and cell invasion (F) assays were performed as described previously. Data are expressed as the mean ± SD of duplicates. The statistical significance was determined using Student's *t* test. *, **, and *** indicate $P < 0.05$, $P < 0.01$, and $P < 0.001$, respectively.

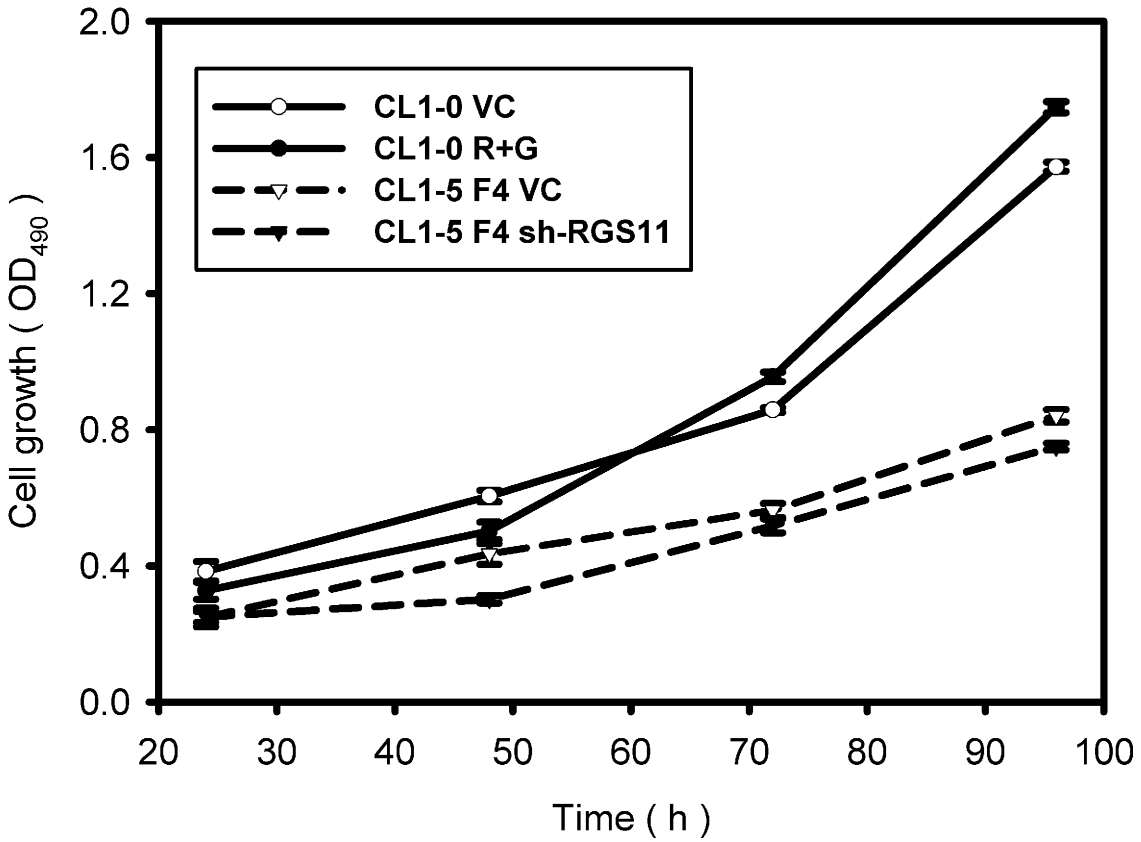


**Supplementary Figure S5: Effect of RGS11 expression level on cell growth.** Poorly-invasive CL1-0 cells were stably cotransfected with plasmids encoding for RGS11 and GNB5 (R+G) to increase their gene expression. On the other hand, highly-invasive CL1-5F4 stable transfectants with attenuated expression of RGS11 were established by transfecting small hairpin interfering RNA specific to RGS11 (sh-RGS11). The control cells, CL1-0 VC and CL1-5F4 VC, were generated by transfecting with pcDNA3.1 and pLKO.1 vectors, respectively. Cells were seeded onto 96-well plates in triplicate and incubated for 24, 48, 72, or 96 h, as indicated. Cell growth of the transfectants was measured by a standard MTT assay. Data are expressed as the mean ± SD of triplicates at $OD_{490}$.

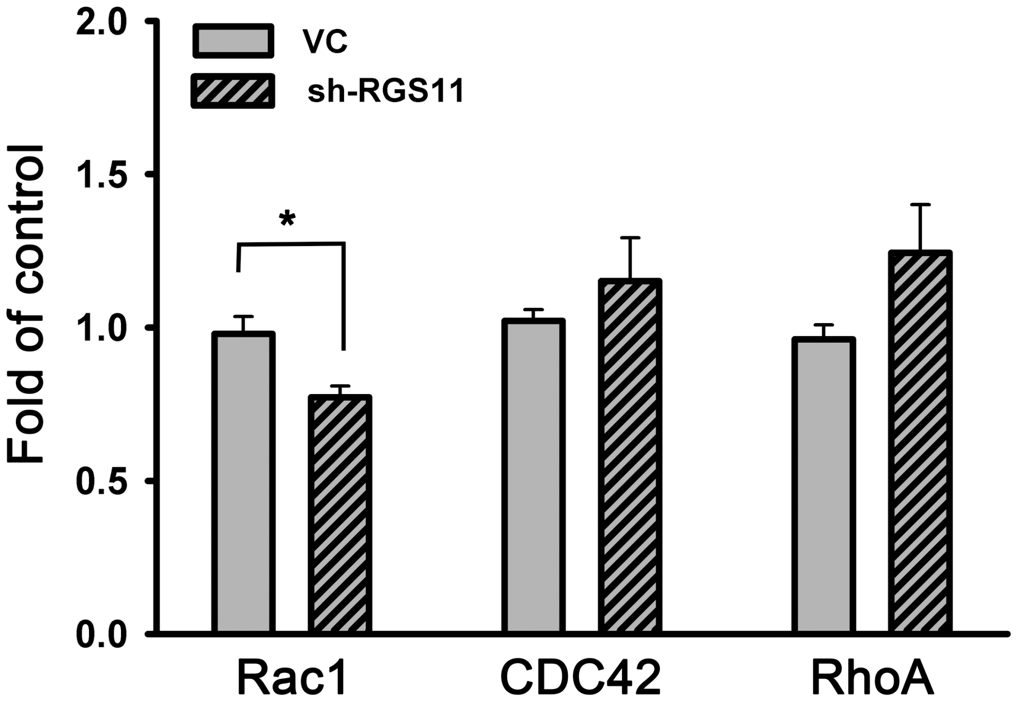


**Supplementary Figure S6: Attenuation of RGS11 impaired the Rac1 activity.** CL1-5F4 stable transfectants with attenuated expression of RGS11 (sh-RGS11) and their vector control (VC) cells were lysed. The GTP-bound RhoA, Rac1, and CDC42 of the lysates (100 µg/sample) were analyzed by binding to their corresponding GTP-binding proteins in G-LISA assay (referring to "Materials and Methods" section for detail). The complex was detected with antibodies specific to the active forms using the absorbance-based G-LISA system (Cytoskeleton) at 490 nm. The experiments were performed in triplicate, and the data are expressed as the mean ± SD fold increase compared with the control cells (VC). * indicates $P < 0.05$.
